# Assessment of the Occurrence of Concentrations of Selected Chemical Elements in Three Types of Cheese from the Retail Chains and Health Risk of Benefits Assessment of Its Consumption

**DOI:** 10.3390/foods15122143

**Published:** 2026-06-14

**Authors:** Martina Pšenková, Róbert Toman, Ivona Jančo

**Affiliations:** 1 Faculty of Agrobiology and Food Resources, Institute of Animal Husbandry, Slovak University of Agriculture in Nitra, Tr. A. Hlinku 2, 949 76 Nitra, Slovakia; martina.psenkova@uniag.sk; 2 AgroBioTech Research Center, Slovak University of Agriculture in Nitra, Tr. A. Hlinku 2, 949 76 Nitra, Slovakia; ivona.janco@uniag.sk

**Keywords:** cheese, essential elements, potentially toxic elements, risk assessment, recommended dietary requirements, food safety

## Abstract

The study aimed to evaluate the nutritional contribution of essential elements (Ca, Fe, K, Mg, Zn) and assess health risks associated with potentially toxic elements (Cd, Pb, As, Al, and Sr) in three types of cheese from retail chains and produced by two different producers. One hundred forty-four cheese samples were collected in 12 months. All samples were analyzed using inductively coupled plasma optical emission spectrometry (ICP-OES). The average concentrations of Ca, Fe, K, Mg, Zn, Al and Sr in cheese samples were 4183.87–6227.98 mg/kg, 1.00–1.63 mg/kg, 695.90–884.39 mg/kg, 175.00–255.70 mg/kg, 21.49–27.56 mg/kg, 2.65–5.73 mg/kg and 1.91–5.82, respectively, depending on cheese type and producers. Concentrations of As, Cd and Pb in all analyzed samples were below the limit of detection (LOD). From a nutritional perspective, the analyzed cheeses represented important dietary sources of calcium and zinc, with substantial contributions to recommended daily intakes, particularly under the higher consumption scenario (100 g/day). Magnesium also contributed meaningfully to dietary intake, especially in children, whereas the contributions of iron and potassium remained relatively low in all evaluated consumption scenarios. Health risk assessment was expressed as the percentage of the tolerable daily intake (%TDI) or provisional tolerable weekly intake (%PTWI). Under the higher consumption scenario (100 g/day), children represented the most highly exposed population group, with %PTWI values for aluminum ranging from 0.25 to 9.10% and %TDI values for strontium ranging from 6.92 to 20.00%, depending on cheese type and producer. Overall, the analyzed cheeses showed high nutritional value and low toxicological risk; however, continuous monitoring of potentially toxic elements remains important to ensure food safety.

## 1. Introduction

Milk and dairy products are considered essential components of the human diet because of their high nutritional value and contribution to daily nutrient intake. Their consumption differs considerably among countries and regions, depending on cultural traditions, geographic conditions, and socio-economic factors [1]. Milk naturally contains a wide range of microminerals and trace elements, including iron (Fe), zinc (Zn), copper (Cu), manganese (Mn), and selenium (Se), which are required in small amounts for the maintenance of normal physiological functions. Other elements, such as chromium (Cr) and nickel (Ni), are generally not regarded as essential for human health [2,3]. Besides its nutritional importance, milk may also represent a source of undesirable and potentially toxic elements, including aluminum (Al), arsenic (As), cadmium (Cd), and lead (Pb). Long-term exposure to these contaminants has been linked with numerous adverse health effects, such as impairment of kidney and liver function, skeletal disorders, neurological damage, endocrine disturbances, reproductive dysfunction, and metabolic abnormalities [4,5,6]. Although the concentrations of highly toxic elements in raw milk and dairy products are usually low, these substances can accumulate in environmental compartments such as soil and water and subsequently enter the food chain, posing risks to both animal and human health [6,7]. For this reason, the determination of toxic element concentrations in milk and dairy products has become an important aspect of food quality control and consumer safety assessment. Regulatory authorities have therefore established monitoring systems and legislative limits for selected contaminants, particularly within the European Union [8]. Nevertheless, several investigations conducted in European countries, including Croatia, Poland, and Serbia, have reported Pb concentrations exceeding the maximum levels permitted by EU legislation [9,10,11].

Certain trace elements, including Cu, Fe, Se, and Zn, are indispensable for biological processes; however, excessive intake may also result in toxicological effects [6]. Elevated copper exposure has been associated with disorders such as Wilson’s disease, characterized by abnormalities in ceruloplasmin metabolism [7]. Similarly, prolonged zinc intake above recommended levels may contribute to severe neurological complications related to copper deficiency [12]. Excess iron intake may also induce toxic effects, particularly in the liver and cardiovascular system, while high iron concentrations can interfere with the absorption and metabolism of other essential elements such as Cu and Zn [13,14]. The occurrence and accumulation of metals in the environment are influenced not only by natural geological conditions but also by human activities. Industrial emissions, mining operations, wastewater disposal, traffic-related pollution, and intensive agricultural practices contribute substantially to environmental contamination [3,15]. These contaminants may subsequently be transferred into products of animal origin, with the level of human exposure depending mainly on dietary habits and consumption frequency. The concentrations of elements in raw milk and dairy products are affected by numerous factors, including feed composition, drinking water quality, animal species, climatic conditions, seasonal changes, and technological processing methods [2,16,17]. Previous studies have also demonstrated considerable differences in the levels of Al, Cd, Cu, Fe, Ni, Pb, and Zn between products originating from industrially polluted and environmentally clean areas, as well as between urban and rural regions [18,19,20,21]. Strontium (Sr) belongs to the same group of elements as calcium (Ca) and behaves very similarly in the body. Plants absorb it from the soil, cows take it in through feed, and some is then excreted in milk [22]. As reported by Nabrzyski and Gajewska [23], for fermented dairy products (yogurts, sour milk products), Sr concentrations can range approximately in the range of 0.21–0.79 mg Sr/kg and an average of 0.44 mg Sr/kg. Strontium behaves similarly to calcium, tending to bind to casein micelles, the solid matrix of cheese, during milk coagulation. As a result, strontium is more concentrated in cheese than in whey during cheesemaking. Studies tracking the distribution of strontium during cheesemaking have shown a strong correlation between the calcium content of cheese and strontium retention, and therefore, soft cheeses with lower mineral content may contain less Sr, while hard and highly mineralized cheeses may show a higher concentration of Sr per kilogram of product [24].

Despite the increasing consumer demand for dairy products and growing concerns regarding contamination by toxic elements, information on the occurrence of essential and toxic elements in cheeses available from the commercial market remains limited. In addition, data evaluating potential dietary exposure and associated health risks related to cheese consumption, particularly among vulnerable population groups such as children, are still insufficient. Therefore, the aim of this study was to determine the concentrations of essential (Ca, Fe, K, Mg, Zn) and toxic elements (Al, Sr, As, Cd, Pb) in cheeses obtained from retail chains, to evaluate dietary intake as well as potential health risks associated with their consumption and to evaluate the nutritional contribution of selected essential elements and the potential health risks associated with exposure to selected potentially toxic elements through cheese consumption in different consumption scenarios.

## 2. Materials and Methods

### 2.1. Sample Collections

Samples of three types of cheese were obtained from retail chains in Slovakia. Cheese 1 was Eidam cheese. Eidam is a semi-hard to hard ripening cheese made from cow’s milk using rennet coagulation. It is produced in several variants according to fat content, most often 30% fat in the curd and 45% fat in the dry matter. The water content in this type of cheese is approximately 40–50% and decreases during ripening, which affects the hardness and slicing of the cheese. Cheese 2 was Polooštiepok cheese. Polooštiepok is a traditional Slovak steamed cheese made most often from cow’s milk (or a mixture of cow’s, sheep’s or goat’s milk). It is a semi-hard steamed cheese and differs from the classic oštiepok mainly in its smaller size and often shorter maturation period. The water content of this cheese is approximately 45–50%, the fat content in dry matter is 40–50%, and the amount of dry matter is approximately 45–50%. Cheese 3 was Parenica cheese. Parenica is a traditional Slovak steamed cheese, most often made from cow’s milk, or a mixture with sheep’s milk. It is a fibrous steamed cheese (pasta filata), similar to Mozzarella. It is characterized by its twisted spiral shape and the possibility of smoked and unsmoked variants. Parenica is made by curdling milk, fermenting the curd, steaming in hot water, stretching the cheese mass into strips, shaping it into a spiral, salting it and possibly smoking it. The water content in this type of cheese is approximately 48–58%, the dry matter content is 42–52% and the fat content in the dry matter is 40–50%.

A total of 144 cheese samples were collected from retail chains during a 12-month monitoring period. The study included three cheese types (Eidam, Polooštiepok and Parenica) obtained from two different producers. Sampling was performed twice per month, at the beginning and at the end of each month. During each sampling event, one sample of each cheese type from each producer was purchased, resulting in six samples per sampling event (3 cheese types × 2 producers). Over the 12-month period, a total of 144 individual cheese samples were collected and analyzed (3 cheese types × 2 producers × 2 sampling events per month × 12 months). Each analytical sample represented one individual commercially packaged cheese product purchased from a retail chain. Samples were analyzed individually and were not pooled prior to analysis. After obtaining product samples, the cheese samples were stored in small plastic boxes at a temperature of −18 °C until laboratory analyses were performed.

### 2.2. Sample Preparations

Before laboratory analysis, all frozen cheese samples were first thawed under controlled conditions at room temperature and subsequently mechanically homogenized using a high-performance disperser Heidolph DIAX 600 homogenizer (Heidolph Elektro GmbH & Co. KG, Schwabach, Germany). Homogenization, as a pre-analytical step, was carried out initially for all samples. The mass of the samples analyzed ranged between 1.0 and 2.0 g and was considered during measurements. Sampling and storage procedures were optimized to minimize potential contamination, losses, or alterations that could adversely affect data reliability. During preparation, only plastic tools for handling and plastic tubes for storage were used.

### 2.3. Analysis of Samples

The presence of 10 elements—essential elements and elements with potential adverse effects on human health (Ca, Fe, K, Mg, Zn, Cd, Pb, As, Al, Sr)—was evaluated using the following methodology. Elemental analysis was performed by inductively coupled plasma optical emission spectrometry (ICP-OES) with an axial plasma configuration and an SPS-3 autosampler.

Initially, the samples underwent mineralization in a high-performance microwave digestion system (Ethos UP, Milestone Srl, Sorisole, Italy) using a mixture containing 5 mL of HNO_3_ (≥69.0%, TraceSELECT^®^, Honeywell Fluka, USA). Additionally, 1 mL of H_2_O_2_ (≥30%, Sigma-Aldrich, Saint Louis, MO, USA) and 2 mL of ultrapure water (18.2 MΩ·cm^−1^ at 25 °C; Synergy UV, Merck Millipore, Guyancourt, France) were added for trace analysis. The digestion procedure consisted of heating and cooling phases. During the heating stage, the samples were warmed for 15 min to 200 °C and this temperature was maintained for another 15 min. Afterward, during the cooling phase, the samples underwent 15 min of active cooling to reach the temperature of 50 °C. The digestates were filtered through the VWR Quantitative filter paper 454 (particle retention 12–15 μm) (VWR International, Leuven, France) into the volumetric flasks and filled up with ultrapure water to a volume of 50 mL.

Subsequently, elemental determination was carried out using an ICP-OES spectrometer (ICP OES 720, Agilent Technologies, MULGRAVE Victoria, Australia) equipped with an axial plasma setup and an SPS-3 autosampler.

For quantitative determination, external calibration was performed using calibration solutions prepared from certified single-element standard solutions. Calibration curves were established using standard solutions in the range of 0.05–10 mg/L. Due to the higher concentrations of macroelements (particularly Ca, K and Mg), additional dilutions of the digested solutions were performed when necessary to ensure that the measured concentrations were within the linear range of the calibration. The final concentrations were recalculated according to the corresponding dilution factors. All samples were analyzed against procedural blanks prepared and processed under identical digestion conditions as the samples. After microwave digestion, the digestates were filtered through VWR Quantitative filter paper 454 (particle retention 12–15 μm), quantitatively transferred into volumetric flasks, and diluted to a final volume of 50 mL with ultrapure water.

Method accuracy was verified using certified reference materials (ERM-BD151 and BCR-274), and recoveries ranged from 90 to 110%. Measurement repeatability was assessed by triplicate analysis of samples. The expanded measurement uncertainty of the analytical method was estimated at 10%.

Quality assurance and quality control procedures included the analysis of procedural blanks and regular measurements of calibration standards throughout the analytical sequence to verify instrument stability and calibration performance. No significant instrumental drift was observed during the analytical runs.

Detection limits (LOD) (mg·kg^−1^) for the analyzed elements were Ca 0.01, Fe 0.1, Mg 0.01, K 0.30, Zn 0.0002, Al 0.0002, As 0.0015, Cd 0.00005, Pb 0.0008, and Sr 0.00001. Quantification limits (LOQ) (μg/kg) for analyzed elements were Ca 0.03, Fe 0.33, Mg 0.03, K 1.00, Zn 0.65, Al 0.67, As 5.0, Cd 0.17, Pb 2.67, and Sr 0.03.

The corresponding analytical wavelengths (nm) were Al 167.019, As 188.980, Cd 226.502, Pb 220.353, Fe 234.350, Sr 407.771, Zn 206.200, Ca 315.887, Mg 383.829 and K 766.491. Detailed instrumental operating parameters are provided in Table 1.

### 2.4. Calculation of Nutritional Coverage for Essential Elements

To assess the role of cheese in daily nutrition, the proportion of recommended daily intake (RDI) for calcium (Ca), magnesium (Mg), iron (Fe), potassium (K), and zinc (Zn) was estimated by assuming a daily consumption of cheese in two different scenarios. For the calculations in the first scenario, data from the Statistical Office of the Slovak Republic in 2024 were used, where the annual consumption of hard and semi-hard cheeses was 4.3 kg per person per year, which corresponds to a daily consumption of cheese of 0.012 kg (12 g). In the case of soft cheeses, the annual consumption was 3.8 kg per person per year, which corresponds to a daily consumption of 0.0104 kg (10 g) [26]. For calculations in the second scenario, the amount of the consumed portion was increased to the recommended nutritional level (0.1 kg–100 g of cheese). The evaluation was based on nutritional guidelines for the Slovak population issued by the Ministry of Health of the Slovak Republic and included different age and physiological groups such as preschool children (4–6 year old) and adult groups with the highest needs (Ca and K for breastfeeding women, Mg and Zn for men with physically hard work, Fe for pregnant women in the second trimester). The study applied the official Slovak age-group classification for nutrient intake recommendations [27]. The document of recommended dietary intakes of the Slovak Republic lacks data on the recommended amount of potassium for population groups per day; therefore, for the calculation of this element, we used the values given by the authors Strohm et al. [28] in Germany.

The percentage contribution of cheese to the Recommended Daily intake (*RDI)* was calculated according to the following equation:

$$\%\;RDI=\frac{C\;\times \;W}{RDI}$$

where C is element concentration in cheese (mg/kg), W is amount of cheese (kg)—depending on scenarios: 1st scenario—cheese consumption per person per day in 2024 for cheese 1 (eidam cheese) and cheese 2 (polooštiepok cheese)—0.012 kg, cheese 3 (parenica cheese) 0.010 kg; 2nd scenario—0.1 kg consumption of cheese and RDI is recommended daily intake for each age group—for preschool children and for age group adults with highest needs (Ca—breastfeeding women; Fe—pregnant women in 2nd trimester; K—breastfeeding women; Mg and Zn—hard working men).

The calculated values were applied to assess the relative significance of cheese as a mineral source among different population groups. To interpret the findings, the contribution of each cheese type to daily mineral intake was classified according to the criteria established in Regulation (EU) No. 1169/2011 of the European Parliament and of the Council [29]. Under this regulation, food can be regarded as a source of minerals when it provides at least 15% of the reference nutrient intake (RDA) per 100 g (European Parliament and Council, 2011). Based on this framework, three levels of contribution were defined: adequate coverage (≥15% of the RDA), intermediate coverage (10–14.9% of the RDA), and insufficient coverage (<10% of the RDA).

Adequate coverage suggests that cheese constitutes a meaningful dietary source of the given mineral, while intermediate or insufficient coverage indicates that additional intake from other foods may be necessary. These findings are particularly relevant for groups with increased mineral demands, such as growing children and pregnant women, for whom sufficient intake of Ca, Mg, K, and Fe is crucial for bone maintenance, proper metabolic functioning, and the prevention of anemia.

### 2.5. Health Risk Assessment

Health risk assessments were performed to evaluate the potential risks associated with the consumption of various cheese types available in retail markets.

A comprehensive risk assessment was conducted to determine the potential health implications of consuming cheese containing aluminum (Al) and strontium (Sr). Arsenic (As), cadmium (Cd), and lead (Pb) concentrations were not detected above the detection limit (LOD) in all analyzed samples; therefore, quantitative risk assessment for these elements could not be performed and were also excluded from the statistical comparison.

The assessment of the intake of toxic elements through the consumption of cheese was estimated to be conducted for two population groups, adults and preschool children, as both groups commonly consume cheese and may therefore represent vulnerable consumer categories [27].

The calculations were based on two consumption scenarios. The first scenario used consumption data provided by the Statistical Office of Slovakia in 2024 [26], while the second considered an increased intake corresponding to recommended nutritional consumption levels.

In the case of the toxic element Al, the level of exposure to this element through cheese consumption, expressed as an estimated daily intake, was expressed through the concentration found in our analyses of cheese samples from individual producers. The calculation was performed using a formula proposed by several authors [7,30].

$$EDI=\frac{\left(C\;\times \;W\right)}{BW}$$

where C (mg/kg) represents the mean level of the monitored element, W represents daily cheese consumption in Slovakia, and BW represents body weight. For an adult person, 70 kg was used in calculations. In the case of children, we also used data from the EFSA Scientific Committee [31], where a weight of 22 kg for children (3–10 years) is listed. According to the Statistical Office of Slovakia in 2024, the mean daily consumption of cheese is 0.012 kg per person in the case of hard cheese and semi-hard (cheese 1 and cheese 2 in this study), and in the case of soft cheese (cheese 3 in this study), 0.010 kg per person per day [26]. For the second scenario of this study, we set the portion of consumption to 0.1 kg of cheese consumed daily.

For aluminum exposure assessment, EDI values were converted to Estimated Weekly Intake (EWI) values [32]:

$$EWI=EDI\;\times \;7\;\left(days\right)$$

Subsequently, these values were used to calculate the percentage contribution to the Provisional Tolerable Weekly Intake (%PTWI) for Al, which was established for this element by JECFA [32]:

$$\%\;PTWI=\frac{EWI}{PTWI}\;\times \;100$$

For strontium (Sr), for which a PTWI value has not been determined, we expressed the proportion (%) of the element intake in the tolerable daily intake (TDI) determined by WHO [33] according to the formula:

$$\%TDI=\frac{EDI}{TDI}\;\times \;100$$

### 2.6. Statistical Analysis

All results of this study were processed using Statistica Cz version 10 (TIBCO Software, Inc., Palo Alto, CA, USA). All obtained results are listed as mean values with standard deviation. Homogeneity of variances was verified using Levene’s test and normality of data using Shapiro–Wilk test. Differences in concentrations of the analyzed elements in different types of cheese between 2 producers were compared by Student’s *t*-test. The level of statistical significance was set at *p* < 0.05.

## 3. Results

### 3.1. Concentrations of Elements in Different Types of Cheese and Producers from Retail Chains

From a nutritional perspective, the elemental composition of different types of cheese from retail chains revealed variation in the concentrations of both essential and potentially toxic elements. The obtained results, summarized in Table 2, indicate that while some elements exhibit relatively stable and similar distributions, others demonstrate pronounced variability, which may be influenced by different factors. Concentrations of essential elements that may become harmful at excessive levels (Ca, Fe, K, Mg, and Zn), as well as toxic elements (Al, As, Pb, Cd, and Sr) found in three types of cheese from different producers, are presented in Table 2. The mean concentrations were detected and are listed in decreasing order as follows: Ca > K > Mg > Zn > Sr > Al > Fe in cheese 1, Ca > K > Mg > Zn > Sr > Fe > Al in cheese 2 and Ca > K > Mg > Zn > Al> Sr > Fe in cheese 3. The other toxic elements such as Cd, Pb, and As were not detected above the limit of detection in any sample cheese from retail chains.

Among the essential elements evaluated, calcium (Ca) exhibited the highest concentration, with an average level from 4183.87 to 6227.98 mg/kg, depending on producers and types of cheese. The detected variability suggests that the Ca content in cheese samples is influenced by several regulatory factors, including nutritional intake of animals, stage of lactation animals where the milk comes on, and technological processing of individual types of cheese. In the case of potassium (K), the highest concentration of this element was recorded in Polooštiepok cheese (cheese 2) from producer 2, with average concentrations of this element ranging from 695.90 to 884.39 mg/kg. The variability of potassium content between individual types of cheese and producers could be largely influenced by the type of cheese, the method of milk processing, the dry matter content and additives, as these factors influence the potassium content in cheeses. Magnesium was the third most abundant essential element in cheese samples, with its average content ranging from 175.00 to 255.7 mg/kg. The lowest concentrations of essential elements were recorded in the case of Zn and Fe, with average Zn values ranging from 21.49 to 27.56 mg/kg, with the lowest Zn content recorded in Polooštiepok cheese (cheese 2) from producer 2. The lowest iron (Fe) content was recorded in Parenica cheese (cheese 3) in both producers, and the same iron concentration of 1.63 mg/kg was recorded in Eidam cheese (cheese 1/producer 2) and Polooštiepok cheese (cheese 2/Producer 1). The differences that were noted in the concentrations of individual cheese types between the two producers were not statistically significant.

In addition to essential elements, several potentially toxic elements were also analyzed in milk samples. Strontium was present in 46 samples analyzed, with average concentration ranging from 1.91 to 5.82 mg/kg. Moreover, the difference in average strontium concentrations in Parenica cheese (cheese 3) was statistically significant (*p* < 0.05) between the producers of this cheese. Strontium is chemically very similar to calcium, so it behaves similarly in soil, water and feed. From feed, it passes into milk, from which cheese is subsequently made. The strontium content in cheese thus directly depends on the geological subsoil of the region and the composition of the feed grazed by the animals, and this fact could have greatly influenced the Sr content in the monitored cheeses. The average Al concentration ranged from 0.16 to 5.73 mg/kg and Al concentration was recorded in 13 monitored cheese samples. In the case of Pološtiepok cheese (cheese 2), we noted a statistically significant difference (*p* ˂ 0.05) in the concentration of Al in samples of this cheese between producers. In the case of Cd, As, and Pb, they were not detected above the limit of detection in any cheese sample.

### 3.2. Contribution of Essential Elements to Nutritional Value

The percentage coverage of the recommended daily intake (RDI) of calcium (Ca), magnesium (Mg), potassium (K), zinc (Zn) and iron (Fe) in different age groups and selected consumption scenarios is shown in Table 3. The findings demonstrate substantial differences among cheese types in their capacity to cover the nutritional requirements for these essential elements across various demographic groups.

The nutritional contribution of the analyzed cheeses was evaluated using Slovak recommended daily intakes for specific population groups. In addition, the results were interpreted in relation to the criteria for nutrition claims established by Regulation (EU) No. 1169/2011. The percentage coverage of Ca ranged from 26.15% to 88.96%, indicating that cheese from this study is a sufficient source of calcium across all studied age groups in the case of scenario 2. The highest calcium coverage was observed among preschool children consuming Eidam cheese (cheese 1) from producer 1 (88.96%), whereas the lowest values were recorded for Polooštiepok cheese (cheese 2) from producer 2 in breastfeeding women (26.15%). Despite the observed decline in calcium coverage with increasing age, all values remained above the 15% threshold, confirming that all monitored sample cheeses from this study can be considered a significant dietary source of calcium for the studied populations. However, if we look at the real consumption of cheese in 2024, i.e., the consumption of cheese according to scenario 1, the percentage coverage of Ca needs in Slovakia in selected age categories in 2024 represented less than 10% of the daily Ca intake, i.e., low coverage, and therefore, it is necessary to increase Ca intake through sources other than cheese.

For Mg, the percentage RDI coverage ranged from 14.58% to 21.31% depending on producers and types of cheese in scenario 2 (100 g/day consumption). These results indicate that magnesium may contribute meaningfully to the daily intake, particularly in children, although its contribution was considerably lower than that of calcium and zinc. Under scenario 1, magnesium coverage remained low in all age categories and did not exceed approximately 6% of the daily requirement.

Zinc was the second most nutritionally relevant element after calcium. Under scenario 2, consumption of 100 g/day of cheese provided 13.43–16.90% of the daily zinc requirement in adults, depending on cheese type and producer. In preschool children, the same consumption scenario provided 46.4–54.0% of the recommended daily zinc intake, indicating that cheese may represent an important dietary source of zinc in this age group.

For the essential elements, Fe and K, adequate coverage of the daily requirement of these elements through cheese consumption was not ensured in either of the selected scenarios. Iron contributed only marginally to the daily requirement, with coverage generally below 2%, even under the higher consumption scenario. Similarly, potassium coverage remained relatively low, reaching a maximum of approximately 6–7% of the daily requirement under scenario 2. Therefore, cheese cannot be considered a significant dietary source of iron or potassium in the studied population groups. Based on these results, adequate intake of Fe and K should be ensured through other food sources.

### 3.3. Health Risk Assessment of Toxic Elements

The evaluation of exposure to aluminum (Al) and strontium (Sr) through the consumption of different types of cheese was performed for various age groups under two daily consumption scenarios (0.012 kg–12 g/day, respectively 0.010 kg–10 g/day and 0.1 kg–100 g/day). The findings, presented in Table 4 and Table 5 depending on age category, show the estimated daily intake (EDI) values for Al and Sr across the examined age categories and demonstrate considerable differences in exposure levels depending on both age and the amount of cheese consumed. For the assessment of aluminum-related health risk, the estimated daily intake (EDI) values were converted to estimated weekly intake (EWI) values according to the equation EWI = EDI × 7. The resulting EWI values were subsequently compared with the provisional tolerable weekly intake (PTWI) of 2 mg/kg body weight/week, and the percentage contribution to PTWI (%PTWI) was calculated accordingly.

The estimated daily intake (EDI) of aluminum was influenced by both the quantity of cheese consumed and the specific producer. In children, EDI values ranged from 0.0012 mg/kg body weight/day at a consumption level of 10 g/day to 0.026 mg/kg body weight/day at 100 g/day. In adults, the estimated intake was lower, varying between 0.00037 and 0.008 mg/kg body weight/day. After conversion of EDI values to estimated weekly intake (EWI), all exposure levels remained substantially below the provisional tolerable weekly intake (PTWI) of 2 mg/kg body weight/week, indicating that cheese consumption alone is unlikely to represent a significant source of toxicological risk associated with aluminum exposure.

The percentage contribution to PTWI (%PTWI) was highest among preschool children aged 4–6 years, ranging from 0.25% to 9.10% depending on the amount and origin of the cheese consumed. In adults, the contribution was considerably lower, with values between 0.01% and 2.80%. Although none of the estimated exposures exceeded the recommended safety threshold, the results suggest that children represent the most highly exposed population group; however, exposure levels remained well below the toxicological reference value. Since aluminum may also be present in other commonly consumed foods and environmental sources, long-term intake from multiple sources could increase overall exposure levels. Consequently, additional assessment of total dietary aluminum intake is advisable, particularly for individuals with frequent cheese consumption and for populations living in areas with elevated environmental aluminum contamination.

The estimated daily intake (EDI) of strontium varied according to both cheese consumption level and producer. In children, the EDI ranged from 0.0009 mg/kg body weight/day at a consumption level of 10–12 g/day to 0.026 mg/kg body weight/day at 100 g/day. Despite these differences, all calculated EDI values remained substantially below the tolerable daily intake (TDI) of 0.13 mg/kg body weight, indicating that cheese consumption alone is unlikely to pose a health risk related to Sr exposure. The contribution of cheese consumption to the percentage of tolerable daily intake (%TDI) was highest in preschool children aged 4–6 years, where values ranged from 0.69% to 20.00% depending on the cheese producer and quantity consumed. In adults, the %TDI values were notably lower, varying between 0.22% and 6.38%. Although the estimated exposures did not exceed the established safety limit, the findings suggest that cheese may still represent a relevant dietary source of Sr, particularly in individuals with frequent or high consumption patterns. Considering that Sr is also present in other commonly consumed foods, such as cereals and leafy vegetables, cumulative dietary exposure may become more significant over time. Continuous intake from multiple dietary sources may therefore contribute to increased body accumulation of this element. For this reason, further assessment of total dietary Sr exposure is recommended, especially in vulnerable population groups and in regions with elevated environmental Sr levels.

## 4. Discussion

### 4.1. Concentrations of Elements in Different Types of Cheese and Producers from Retail Chains

From the nutritional point of view, the elemental profile of milk and dairy products comprises both physiologically indispensable and biologically non-essential elements [34]. Under normal environmental conditions, the concentrations of toxic metals in uncontaminated dairy products remain relatively low. Nevertheless, increasing anthropogenic pressure associated with industrialization, intensive agriculture, and urban development contributes to the release and subsequent bioaccumulation of hazardous substances within environmental compartments and the food chain. The toxicodynamic and toxicokinetic effects of metals are influenced by numerous interacting factors, including individual biological characteristics, exposure pathway, chemical speciation and solubility of the element, magnitude and duration of exposure, frequency of intake, gastrointestinal absorption efficiency, tissue retention capacity, and the effectiveness of detoxification and excretory mechanisms [35,36].

Milk and dairy products are a very good source of minerals. Consumption of dairy products is important, especially in the prevention of osteoporosis and for the proper development of bones and teeth. This study showed that monitored cheese available on the Slovak retail chains are very rich in elements such as Ca, Mg and K. The levels of essential and toxic elements detected in different types of cheese in the present study were generally consistent with data previously published in the scientific literature; however, several differences in element concentrations were also identified. The highest concentrations in the cheese samples were recorded for calcium, the highest concentration of which was recorded in Eidam cheese and Polooštieopok cheese with an average value of 6.227.98 and 5997.43 mg/kg.

In the case of K, the highest concentrations of this element were found in cheese Polooštiepok (cheese 2) and cheese Parenica (cheese 3), where the average concentration ranged between 855.91 and 884.39 mg/kg. On the other hand, the highest Mg concentrations were again found in Eidam cheese, where the average concentration ranged between 255.7 and 237.11 mg/kg depending on the cheese producer. Compared with the results of the study by Capcárová et al. [37], who monitored the content of essential and toxic elements in the dairy product cottage cheese, we recorded significantly higher concentrations of Ca, Mg and K. This fact was probably influenced by the biochemical processes in cheese production. The loss of water content in different stages of hard cheese production is one of the reasons for the assumed higher concentration of trace elements compared to fresh cheese or dairy products that contain more water [38,39].

Zinc was an essential element in our study, and based on its concentration, we found that it was the only one that would cover the recommended daily intake in sufficient quantities based on cheese consumption. Average Zn concentrations ranged from 21.49 to 27.56 mg/kg, depending on the producer and type of cheese. The high concentration of Zn could be explained by the general abundance of this element and the fact that 95% of its total content is retained by casein micelles during cheese production. However, our reported concentrations for Zn in this study were lower than concentrations in Lebanese cheese [40] or As concentrations, which are reported for Italian mozzarella and goat cheese [41,42] or Turkish white cheese [43].

Iron was the least abundant essential element in our study. Average Fe concentrations ranged from 1.0 to 1.63 mg/kg, with the same Fe concentration in the samples of Eidam (cheese 1) and Polooštiepok (cheese 2) cheese, namely a concentration of 1.63 mg/kg. Significantly higher values of Fe concentrations are reported by Šnirc et al. [44], who found through analyses the Fe content at the level of 4.90–26.2 mg/kg in Oštiepok cheese, which is a traditional Slovak cheese and is very similar to Pološtiepok (cheese 2), with the only difference being in the size and shape of the cheese and the technological processing being almost the same.

Environmental pollution, particularly anthropogenic contamination, is considered one of the major factors contributing to the occurrence of trace and potentially toxic elements in food [45]. Among the toxic elements monitored in the present study (Al, As, Pb, Cd, and Sr), the concentrations of cadmium (Cd), arsenic (As), and lead (Pb) in all analyzed cheese samples from both producers were not detected above the limit of detection (LOD). The limits of detection achieved in the present study were 0.0015 mg/kg for As, 0.00005 mg/kg for Cd, and 0.0008 mg/kg for Pb. These values indicate a high analytical sensitivity of the applied ICP-OES method and were sufficiently low for the determination of trace concentrations of these elements in cheese samples. Therefore, the fact that As, Cd, and Pb were not detected above the respective limits of detection suggests that their concentrations in the analyzed cheeses were very low.

Cadmium is recognized as a highly toxic element with adverse effects on human health [46], although milk and dairy products generally contain relatively low concentrations of this contaminant. Nevertheless, as reported by Totan and Filazi [17], elevated concentrations of cadmium and lead have been detected in dairy products originating from regions affected by intensive industrial activity and other anthropogenic pollution sources [47].

From a regulatory perspective, it should be noted that the occurrence of contaminants in food is governed by the current European Union legislative framework. However, specific maximum levels are not established for all analyzed elements in cheese and dairy products. Therefore, in addition to considering available regulatory requirements, the toxicological significance of the monitored elements was evaluated using internationally recognized health-based guidance values. Cadmium has been assessed by JECFA [4] through a provisional tolerable monthly intake (PTMI) of 0.025 mg/kg body weight, while the former provisional tolerable weekly intake (PTWI) established for lead has been withdrawn because no safe exposure threshold could be identified. For arsenic, toxicological evaluation is currently based on risk assessment approaches rather than a universally accepted tolerable intake value. Since Cd, Pb, and As were below the limit of detection in all analyzed samples, these elements do not represent a significant food safety concern in the cheeses investigated in the present study.

Concentrations of Al which were found in cheese samples ranged from 2.93 to 5.73 in Eidam and Parenica cheeses. In the case of aluminum, we noted a statistically significant (*p* ˂ 0.05) difference in Polooštiepok cheese when comparing the concentrations of this element between producers. Aluminum contamination in milk and dairy products may originate from several different sources, including technological processing and storage conditions. A significant contribution to aluminum levels is associated with contact between cheese and aluminum-based equipment or containers used during production and handling processes. The use of aluminum utensils for processing or storing dairy products can therefore markedly increase the concentration of this element in the final products. Considerable variability in aluminum concentrations has been reported in dairy products from Egypt, where measured levels reached 19.93 ppm in raw farm milk, 107.32 ppm in commercially available milk, 52.34 ppm in Kareish cheese, 4.19 ppm in yogurt, and 80.97 ppm in rice pudding [36].

The detected Sr concentrations were from 1.91 mg/kg in cheese Parenica to 5.82 mg/kg in cheese Eidam. When comparing Sr concentrations between producers, we noted a statistically significant difference (*p* ˂ 0.001) in Parenica cheese. Concentrations of Sr in Parenica and Polooštiepok cheese are approximately 50% lower than reported in the work by Almášiová et al. [25], where the Sr concentration in cheese was at the level of 4.96 mg/kg. Strontium acts as a calcium antagonist and could accumulate on bone surfaces during adulthood. In children, however, this element may become incorporated into the developing mineral structure of bones, resulting in its long-term retention within the organism. Insufficient dietary intake of calcium and proteins may further enhance the adverse effects of strontium, potentially leading to impaired skeletal development and reduced bone growth [48].

### 4.2. Contribution of Essential Elements to Nutritional Value

Our results showed that daily consumption of 100 g (scenario 2) of any cheese from this study covers 59.77–88.96% of the RDI for calcium in preschool children aged 4–6 years and, in the case of adults, ranged between 26.15 and 38.92% depending on producers. According to Regulation (EU) No. 1169/2011, foods containing more than 15% of the recommended daily nutrient reference value per 100 g are permitted to bear the nutrition claim “source of calcium” [28]. Nevertheless, calcium requirements substantially increase during adolescence and adulthood, reaching approximately 1200–1500 mg per day. Consequently, maintaining sufficient calcium intake often requires the inclusion of additional dietary sources, besides matured cheeses, also calcium-fortified plant-based beverages, and green leafy vegetables [49].

By analyzing zinc concentrations, we found that zinc was the only element whose RDI monitored through consumption of the cheeses would be ensured in both age categories in consumption of 100 g of any type of cheese and these contribution on RDI was in accordance with Regulation (EU) No. 1169/2011 [28] that foods which contain more than 15% of the recommended daily nutrient reference value per 100 g are a good source of this element. The percentage of contribution for children was from 52% to 54% and for adults, it was from 15.5% to 16.9%.

The results of this study showed that a daily intake of 100 g of any type of cheese covers 15.57–21.31% of RDA for magnesium in preschool children aged 4–6 years depending on producers and in adults’ categories, from 4.17% to 6.09%. In accordance with Regulation (EU) No. 1169/2011, products containing between 15% and 30% of the recommended nutrient intake per 100 g are eligible to be classified as a “moderate source” of a given mineral [28]. From the perspective of children, the cheeses analyzed may represent a better source of Mg to cover the daily requirement compared to adults, and therefore, it is important for adults to include other types of foods such as nuts, seeds, whole grains, and leafy greens in their diet.

In the case of iron, our study found the lowest content of this element in the analyzed cheeses. Consumption of 100 g of any cheese would ensure coverage of the recommended daily intake at the level of 1.1–1.81%, depending on the producer and type of cheese, in the age category of preschool children. In adults, these values would be significantly lower, at the level of 0.062–0.54%. The bioavailability of iron in milk and dairy products is relatively low, primarily due to the dominance of non-heme iron forms and the inhibitory effect of calcium on intestinal iron absorption. Experimental findings reported by Perfecto et al. [50] indicated that the presence of dairy matrices reduced iron transport across Caco-2 cell models by approximately 50%. Although iron associated with lactoferrin may facilitate improved absorption efficiency, this fraction constitutes only a minor proportion of total milk iron, accounting for roughly 10% [51], thereby limiting its physiological significance for overall iron status. In addition, several dietary components substantially influence non-heme iron utilization. Compounds such as phytic acid present in cereals and polyphenols occurring in plant-derived foods have been shown to markedly decrease iron absorption, whereas factors including ascorbic acid and fermentation processes may significantly enhance its bioavailability [52,53].

Our results showed that daily consumption of 100 g of any cheese from this study covers 5.77–6.80% of the RDI for potassium in preschool children aged 4–6 years and in the case of adults, ranged between 1.70 and 1.9% depending on producers. Similarly to iron, in the case of potassium, we can state that the coverage of the RDI based on Regulation (EU) No. 1169/2011 [28], that foods which contain less than 10% of the recommended daily nutrient reference value per 100 g are not good sources of this element. According to current public health evidence, insufficient potassium intake combined with excessive sodium consumption represents a major nutritional factor contributing to the global burden of cardiovascular diseases. Diets dominated by high salt intake and inadequate consumption of potassium-rich foods are commonly associated with elevated blood pressure, increased incidence of stroke, coronary disorders, and higher mortality rates [54,55,56]. For this reason, international health authorities and epidemiological research consistently promote dietary patterns based on healthier lifestyle habits, particularly increased intake of fruits and vegetables, which constitute important natural sources of potassium and contribute to maintaining cardiovascular health [57].

### 4.3. Health Risk Assessment of Toxic Elements

The estimated daily intake of aluminum in this study ranged between 0.0012 and 0.026 mg/kg body weight/day for children and between 0.00037 and 0.008 mg/kg body weight/day for adult age categories depending on the amount of cheese and producers. For the assessment of health risk, EDI values were converted to estimated weekly intake (EWI) values (EWI = EDI × 7), which were subsequently used to calculate the percentage contribution to the provisional tolerable weekly intake (%PTWI). According to calculations, these concentrations represent 0.13–2.87% PTWI for adults and 0.42–9.12% PTWI for children. The provisional tolerable weekly intake (PTWI) value set by JECFA [32] for Al is 2 mg/kg body weight/week. Based on the calculated EWI values, the weekly intakes of Al through cheese in individual age categories are below the set value in both monitored cheese consumption scenarios. Therefore, it can be stated that the consumption of these cheeses is safe for children and adults, also from the point of view that Al was detected in only 11 samples out of the total number of samples analyzed. Despite the low levels of this element found in our analysis, it is necessary to monitor this metal in milk and dairy products, especially in young children and infants, as they are at higher risk of brain and bone damage, especially in children with kidney damage [58].

The estimated daily intake of strontium in this study ranged from 0.0007 to 0.026 mg/kg body weight/day for children and from 0.00029 to 0.0083 mg/kg body weight/day for adult-aged categories. WHO has set the TDI for Sr at 0.13 mg/kg [33]. Likewise, for strontium, we can state that our detected values were in line with this limit and were significantly below this set value. According to calculations, concentrations of EDI represent 0.71–20.31% for children and 0.23–6.38% for adult age categories from the value of TDI for Sr. Strontium may be important in terms of food contamination because, together with Li, it can affect the metabolism of essential Ca and may be a cause of osteomalacia [59]. Neither the European Union nor the Slovak Food Codex SR sets specific maximum permissible levels for stable (non-radioactive) strontium in cheeses. Limits for common heavy metals in dairy products apply to substances such as lead and cadmium. The only legislative limits for strontium concern the radioactive isotope strontium—^90^Sr. These limits are set by Council Regulation (Euratom) 2016/52 [60] in the event of a nuclear accident or radiological emergency. For milk and dairy products, including cheese, the maximum permissible level of radioactive contamination with strontium-90 is set at 125 Bq/kg. In the normal regime, milk and dairy products are controlled in Slovakia with long-term results well below these limits, close to the limit of measurability of the State Veterinary Administration of the Slovak Republic.

## 5. Conclusions

The present study demonstrated that the analyzed cheese samples constitute an important dietary source of essential minerals, particularly calcium and zinc, which contributed substantially to the recommended daily nutrient intakes. Magnesium also provided a meaningful nutritional contribution, especially under the higher consumption scenario and in children. In contrast, the contributions of potassium and iron to the recommended daily intake remained relatively low. The elemental composition varied depending on cheese type and producer, indicating that manufacturing practices and raw material origin may influence mineral content. Regarding food safety, the concentrations of arsenic, cadmium, and lead in all analyzed samples were below the limit of detection, suggesting a low level of contamination by these toxic elements. Although aluminum and strontium were detected in several cheese samples, the estimated dietary exposure generally remained below the established health-based guidance values. However, children may represent a more vulnerable population group, particularly when consuming the recommended daily amount of cheese, due to the relatively higher contribution of aluminum and strontium to tolerable intake limits. Overall, the results confirm that cheese is a nutritionally valuable food, particularly as a source of calcium and zinc, with a relatively low toxicological risk under normal consumption conditions. Nevertheless, continuous monitoring of potentially toxic elements in dairy products remains necessary to ensure consumer safety, especially for sensitive population groups and in view of possible variations associated with production practices and environmental contamination.

## Figures and Tables

**Table 1:** The operating parameters of determination of elements by ICP-OES [25].

Parameter	Value
RF Power [kW]	1.30
Plasma flow [L/min]	15.0
Auxiliary flow [L/min]	1.50
Nebulizer flow [L/min]	0.85
Replicated read time [s]	5.00
Instrument stabilization [s]	15
Sample uptake delay [s]	25
Pump rate [rpm]	15
Rinse time [s]	10

**Table 2:** Mean concentrations with standard deviation of monitored elements in different types of cheese and producers (mg/kg).

	Mean ± SD					
	Cheese 1		Cheese 2		Cheese 3	
	Producer 1	Producer 2	Producer 1	Producer 2	Producer 1	Producer 2
Ca	6227.98 ± 514.32	5868.55 ± 754.65	4921.59 ± 1422.42	4183.87 ± 1551.31	5535.85 ± 717.86	5997.43 ± 436.16
Fe	1.56 ± 0.72	1.63 ± 1.08	1.63 ± 1.39	1.53 ± 1.23	1.28 ± 0.64	1.00 ± 0.67
K	803.69 ± 149.68	695.90 ± 121.95	750.04 ± 124.81	884.39 ± 341.72	786.41 ± 180.42	855.91 ± 121.16
Mg	255.70 ± 29.39	237.11 ± 27.43	198.64 ± 26.82	175.00 ± 38.35	204.99 ± 37.45	230.49 ± 24.03
Zn	26.12 ± 3.51	26.32 ± 4.53	24.78 ± 3.25	21.49 ± 7.44	26.41 ± 3.14	27.56 ± 2.62
Al	2.93 ± 0.03	3.39 ± 0.72	3.11 ± 1.23 ^a^	5.73 ± 0.94 ^b^	0.16 ± 0.15	2.65 ± 0.15
Sr	5.47 ± 8.29	5.82 ± 8.57	2.15 ± 0.23	1.91 ± 0.67	2.05 ± 0.12 ^a^	2.51 ± 0.25 ^b^
Cd	ND					
As	ND					
Pb	ND					

Concentrations within the same cheese type marked with different superscript letters differ significantly (*p* < 0.05); ND—not detected, below LOD; cheese 1—Eidam cheese; cheese 2—polooštiepok cheese; cheese 3—parenica cheese.

**Table 3:** Recommended daily intake of essential elements and contribution (%) of these elements to the recommended daily intake levels for essential nutrients according to consumption of cheese and producers.

Element	Ca	Fe	K	Mg	Zn
Recommended Daily Intake (Children/Group of Adults with the Highest Need) *	700 mg/1600 mg [26]	9 mg/30 mg [26]	1300 mg/4400 mg [27]	120 mg/420 mg [26]	5 mg/16 mg [26]
	Producer 1/Producer 2				
**Consumption of cheese ^1^ 100 g (2nd scenario)**					
% contribution for children	88.96/83.83	1.73/1.81	6.18/5.35	21.31/19.75	52.2/52.4
% contribution for adults	38.92/36.68	0.5/0.53	1.83/1.70	6.09/5.64	16.31/16.37
**Consumption of cheese ^1^ 12 g (1st scenario)**					
% contribution for children	10.68/10.6	0.21/0.22	0.74/0.64	2.56/2.37	6.2/6.4
% contribution for adults	4.67/4.4	0.062/0.064	0.22/0.19	0.73/0.68	1.96/1.97
**Consumption of cheese ^2^ 100 g (2nd scenario)**					
% contribution for children	70.31/59.77	1.78/1.67	5.77/6.80	16.55/14.58	49.4/46.4
% contribution for adults	30.76/26.15	0.53/0.51	1.70/2.00	4.72/4.17	15.5/13.43
**Consumption of cheese ^2^ 12 g (1st scenario)**					
% contribution for children	8.44/7.17	0.2/0.21	0.69/0.82	1.98/1.75	5.8/5.2
% contribution for adults	3.69/3.14	0.06/0.063	0.20/0.24	0.57/0.5	1.81/1.62
**Consumption of cheese ^3^ 100 g (2nd scenario)**					
% contribution for children	79.1/85.7	1.81/1.1	6.08/6.6	17.00/19.2	52.00/54.00
% contribution for adults	34.6/37.5	0.54/0.3	1.8/1.9	4.7/5.5	16.3/16.9
**Consumption of cheese ^3^ 10 g (1st scenario)**					
% contribution for children	7.91/8.57	0.142/0.11	0.6/0.66	1.7/1.92	5.2/5.4
% contribution for adults	3.46/3.75	0.043/0.033	0.18/0.19	0.47/0.55	1.63/1.69

* Explained in the methods; Cheese ^1^—Eidam cheese; Cheese ^2^—polooštiepok cheese; Cheese ^3^—parenica cheese.

**Table 4:** Estimated daily intakes of elements for children based on different levels of cheese consumption, compared with the corresponding TDI, PTWI, and values listed in the table for each element.

Element	EDI (mg/kg) Cheese 1 100 g	EDI (mg/kg) Cheese 1 12 g	EDI (mg/kg) Cheese 2 100 g	EDI (mg/kg) Cheese 2 12 g	EDI (mg/kg) Cheese 3 100 g	EDI (mg/kg) Cheese 3 10 g	% of Applicable Limit in Cheese ^1^		% of Applicable Limit in Cheese ^2^		% of Applicable Limit in Cheese ^3^	
							100 g	12 g	100 g	12 g	100 g	10 g
	Producer 1/Producer 2						Producer 1/Producer 2					
Al ^a^	0.013/0.015	0.0015/0.0018	0.014/0.026	0.0016/0.003	0.0007/0.012	0.00007/0.0012	4.55/5.25	0.53/0.63	4.90/9.10	0.56/1.05	0.25/4.20	0.02/0.42
Sr ^b^	0.025/0.026	0.003/0.0032	0.009/0.02	0.0012/0.0021	0.009/0.011	0.0009/0.00114	19.23/20.00	2.31/2.46	6.92/15.38	0.92/1.62	6.92/8.46	0.69/0.88
Cd	ND											
Pb	ND											
As	ND											

^1^—Eidam cheese; ^2^—Polooštiepok cheese; ^3^—Parenica cheese; ^a^ 2 mg/kg PTWI [32]; ^b^ 0.13 mg/kg TDI [33]; EDI—estimated daily intake.

**Table 5:** Estimated daily intakes of elements for adults based on different levels of cheese consumption, compared with the corresponding TDI, PTWI, and values listed in the table for each element.

Element	EDI (mg/kg) Cheese 1 100 g	EDI (mg/kg) Cheese 1 12 g	EDI (mg/kg) Cheese 2 100 g	EDI (mg/kg) Cheese 2 12 g	EDI (mg/kg) Cheese 3 100 g	EDI (mg/kg) Cheese 3 10 g	% of Applicable Limit in Cheese ^1^		% of Applicable Limit in Cheese ^2^		% of Applicable Limit in Cheese ^3^	
							100 g	12 g	100 g	12 g	100 g	10 g
	Producer 1/Producer 2						Producer 1/Producer 2					
Al ^a^	0.004/0.005	0.0005/0.0006	0.0044/0.008	0.0005/0.0009	0.0002/0.004	0.00002/0.0004	1.40/1.75	0.18/0.21	1.54/2.80	0.18/0.32	0.07/1.40	0.007/0.14
Sr ^b^	0.008/0.0083	0.0009/0.00099	0.003/0.0056	0.0004/0.0007	0.0029/0.0035	0.00029/0.00035	6.15/6.38	0.69/0.76	2.31/4.31	0.31/0.54	2.23/2.69	0.22/0.27
Cd	ND											
Pb	ND											
As	ND											

^1^—Eidam cheese; ^2^—Polooštiepok cheese; ^3^—Parenica cheese; ^a^ 2 mg/kg PTWI [32]; ^b^ 0.13 mg/kg TDI [33]; EDI—estimated daily intake.

## Data Availability

The raw data supporting the conclusions of this article will be made available by the authors on request.
